# Correlating Reiff scores with clinical, functional, and prognostic factors: characterizing noncystic fibrosis bronchiectasis severity: validation from a nationwide multicenter study in Taiwan

**DOI:** 10.1186/s40001-024-01870-z

**Published:** 2024-05-14

**Authors:** Wen-Chien Cheng, Chia-Ling Chang, Chau-Chyun Sheu, Ping-Huai Wang, Meng-heng Hsieh, Ming-Tsung Chen, Wei-Fan Ou, Yu-Feng Wei, Tsung-Ming Yang, Chou-Chin Lan, Cheng-Yi Wang, Chih-Bin Lin, Ming-Shian Lin, Yao-Tung Wang, Ching-Hsiung Lin, Shih-Feng Liu, Meng-Hsuan Cheng, Yen-Fu Chen, Chung-Kan Peng, Ming-Cheng Chan, Ching-Yi Chen, Lun-Yu Jao, Ya-Hui Wang, Chi-Jui Chen, Shih-Pin Chen, Yi-Hsuan Tsai, Shih-Lung Cheng, Horng-Chyuan Lin, Jung-Yien Chien, Hao-Chien Wang, Wu-Huei Hsu, Taiwan Bronchiectasis Research Collaboration (TBARC)

**Affiliations:** 1 Division of Pulmonary and Critical Care Medicine, Department of Internal Medicine, China Medical University Hospital, Taichung, Taiwan; 2 Critical Medical Center, China Medical University Hospital, Taichung, Taiwan; 3 School of Medicine, College of Medicine, China Medical University, Taichung, Taiwan; 4 Department of Internal Medicine, National Taiwan University Hospital Hsin-Chu Branch, Hsin-Chu, Taiwan; 5 Graduate Institute of Clinical Medicine, College of Medicine, National Taiwan University, Taipei, Taiwan; 6 Division of Pulmonary and Critical Care Medicine, Department of Internal Medicine, Kaohsiung Medical University Hospital, Kaohsiung, Taiwan; 7 Department of Internal Medicine, School of Medicine, College of Medicine, Kaohsiung Medical University, Kaohsiung, Taiwan; 8 Division of Thoracic Medicine, Far Eastern Memorial Hospital, Taipei, Taiwan; 9 Department of Thoracic Medicine, Chang Gung Memorial Hospital at Linkou, Taoyuan, Taiwan; 10 College of Medicine, Chang Gung University, Taoyuan, Taiwan; 11 Division of Pulmonary and Critical Care Medicine, Department of Internal Medicine, Tri-Service General Hospital, National Defense Medical Center, Taipei, Taiwan; 12 Division of Chest Medicine, Department of Internal Medicine, Taichung Veterans General Hospital, Taichung, Taiwan; 13 Department of Internal Medicine, E-Da Cancer Hospital, I-Shou University, Kaohsiung, Taiwan; 14 School of Medicine for International Students, College of Medicine, I-Shou University, Kaohsiung, Taiwan; 15 Division of Pulmonary and Critical Care Medicine, Chiayi Chang Gung Memorial Hospital, Chiayi, Taiwan; 16 Division of Pulmonary Medicine, Department of Internal Medicine, Taipei Tzu Chi Hospital, Buddhist Tzu Chi Medical Foundation, New Taipei City, Taiwan, Republic of China; 17 Department of Internal Medicine, Cardinal Tien Hospital and School of Medicine, College of Medicine, Fu Jen Catholic University, New Taipei City, Taiwan; 18 Division of Pulmonary Medicine, Department of Internal Medicine, Hualien Tzu Chi Hospital, Buddhist Tzu Chi Medical Foundation, Hualien, Taiwan; 19 School of Medicine, Tzu-Chi University, Hualien, Taiwan; 20 Department of Respiratory Care, Chang Gung University of Science and Technology, Chiayi, 613016 Taiwan; 21 Division of Pulmonary Medicine, Department of Internal Medicine, Chung Shan Medical University Hospital, Taichung, Taiwan; 22 School of Medicine, Chung Shan Medical University, Taichung, Taiwan; 23 Department of Internal Medicine, Division of Chest Medicine, Changhua Christian Hospital, Changhua, Taiwan; 24 Institute of Genomics and Bioinformatics, National Chung Hsing University, Taichung, Taiwan; 25 Ph.D. Program in Translational Medicine, National Chung Hsing University, Taichung, Taiwan; 26 Department of Recreation and Holistic Wellness, MingDao University, Changhua, Taiwan; 27 Division of Pulmonary & Critical Care Medicine, Department of Internal Medicine, Kaohsiung Chang Gung Memorial Hospital, Kaohsiung, Taiwan; 28 Department of Respiratory Therapy, Kaohsiung Chang Gung Memorial Hospital, Kaohsiung, Taiwan; 29 Chang Gung University College of Medicine, Taoyuan, Taiwan; 30 Department of Respiratory Therapy, College of Medicine, Kaohsiung Medical University, Kaohsiung, Taiwan; 31 Department of Internal Medicine, National Taiwan University Hospital Yun-Lin Branch, Yun-LIn, Taiwan; 32 Thoracic Medicine Center, Department of Medicine and Surgery, National Taiwan University, Taipei, Taiwan; 33 Department of Medical Planning, Medical Affairs Bureau Ministry of National Defense, Taipei, Taiwan; 34 Department of Critical Care Medicine, Taichung Veterans General Hospital, Taichung, Taiwan; 35 School of Post Baccalaureate Medicine, College of Medicine National Chung Hsing University, Taichung, Taiwan; 36 School of Medicine, Tzu-Chi University, Hualien, Taiwan, Republic of China; 37 Medical Research Center, Cardinal Tien Hospital and School of Medicine, College of Medicine, Fu Jen Catholic University, New Taipei City, Taiwan; 38 Department of Pulmonary Medicine, Lee’s Clinic, Pingtung, Taiwan; 39 Department of Respiratory Therapy, Chang Gung Memorial Hospital at Linkou, Taoyuan, Taiwan; 40 Department of Internal Medicine, National Taiwan University Hospital, National Taiwan University College of Medicine, Taipei, Taiwan; 41 Department of Medicine, National Taiwan University Cancer Center, National Taiwan University College of Medicine, Taipei, Taiwan

## Abstract

**Background:**

Our study aimed to confirm a simplified radiological scoring system, derived from a modified Reiff score, to evaluate its relationship with clinical symptoms and predictive outcomes in Taiwanese patients with noncystic fibrosis bronchiectasis (NCFB).

**Methods:**

This extensive multicenter retrospective study, performed in Taiwan, concentrated on patients diagnosed with NCFB verified through high-resolution computed tomography (HRCT) scans. We not only compared the clinical features of various types of bronchiectasis (cylindrical, varicose, and cystic). Furthermore, we established relationships between the severity of clinical factors, including symptom scores, pulmonary function, pseudomonas aeruginosa colonization, exacerbation and admission rates, and HRCT parameters using modified Reiff scores.

**Results:**

Data from 2,753 patients were classified based on HRCT patterns (cylindrical, varicose, and cystic) and severity, assessed by modified Reiff scores (mild, moderate, and severe). With increasing HRCT severity, a significant correlation was found with decreased forced expiratory volume in the first second (FEV1) (*p* < 0.001), heightened clinical symptoms (*p* < 0.001), elevated pathogen colonization (pseudomonas aeruginosa) (*p* < 0.001), and an increased annual hospitalization rate (*p* < 0.001). In the following multivariate analysis, elderly age, pseudomonas aeruginosa pneumonia, and hospitalizations per year emerged as the only independent predictors of mortality.

**Conclusion:**

Based on our large cohort study, the simplified CT scoring system (Reiff score) can serve as a useful adjunct to clinical factors in predicting disease severity and prognosis among Taiwanese patients with NCFB.

## Introduction

Bronchiectasis is identified by persistent abnormal enlargement of an airway [1]. Precise diagnosis of bronchiectasis is achieved using high-resolution computed tomography (HRCT), which allows for quantitative assessment of morphological changes related to the condition [2]. There are two main types of bronchiectasis: noncystic fibrosis bronchiectasis (NCFB) and cystic fibrosis bronchiectasis (CFB). CFB, a genetic condition, results in the accumulation of thick mucus buildup in the lungs and other organs, causing respiratory challenges and lung damage [3]. NCFB is known for its heterogeneity, with symptoms and pulmonary damage varying widely in severity [3, 4]. Over the years, several radiological scoring systems have been proposed to evaluate the severity of the disease.

Bhalla et al. created a comprehensive scoring system to measure structural lung abnormalities in 14 patients with CFB using thin-section CT scans [5]. Reiff et al. presented a scoring system encompassing both CFB and NCFB, outlining the site, type, and extent of bronchiectasis [6]. Bedi et al. introduced the Bronchiectasis Radiologically Indexed CT Score (BRICS), which combines bronchial dilation and emphysema-affected segments on CT scans. BRICS correlated significantly with clinic prognostic markers, providing a useful tool for evaluating NCFB in clinical practice [7]. There were some limitations to these scoring systems. The Bhalla score, initially developed for patients with CFB, has been expanded to include all bronchiectasis types, but its complexity limits its clinical utility [8]. The Reiff score, based on subjective evaluation of bronchiectasis features, fails to capture the diversity of the disease. It has the potential to assign high scores to patients with localized but severe abnormalities while neglecting those with widespread yet less pronounced structural problems [9]. While BRICS considers clinic prognostic factors, it lacks a relationship between specific CT features and disease activity degree, a vital factor in treatment decisions [7]. These scoring systems lack integration with clinical parameters, which is a significant drawback. Moreover, all these scoring systems are based on western populations to evaluate the severity of bronchiectasis.

Among these score systems, modified Reiff scores have been extensively used in studies [6]. However, a few studies have comprehensively correlated modified Reiff scores with clinical parameters in East Asian patients with NCFB. As a result, we performed a multicenter study in Taiwan, including patients from northern, central, southern, and eastern regions, without limiting to specific causes of NCFB. Our aim was not only to confirm the association between the modified Reiff score and clinical prognostic factors but also to analyze the clinical characteristics of patients with NCFB based on bronchiectasis patterns (cylindrical, varicose, and cystic).

## Methods

### Study setting and participants

The Taiwan Bronchiectasis Registry is a multicenter, retrospective, observational cohort study. We performed a retrospective review of medical records from 16 healthcare sites across Taiwan, involving patients diagnosed with NCFB based on the 2017 European Respiratory Society guidelines[10] between January 2017 and June 2020. Excluding individuals aged less than 20 years, and those with less than one year of follow-up, the study cohort consisted of 2753 patients. The study protocol was approved by the Institutional Review Board of each site and followed the amended Declaration of Helsinki. Patient information was anonymized and deidentified before analysis, eliminating the requirement for informed consent due to the retrospective nature of the study.

### Collection of clinical and demographics data

Upon enrollment, detailed medical records were gathered, including demographics, clinical manifestations, comorbidities, lung function, exacerbations, microbiology, and radiology. A 1-year follow-up involved tracking acute exacerbation frequency and monitoring survival status after enrollment. CT scans were assessed collaboratively by a pulmonologist and a radiologist, with the date of CT serving as the index date. Discrepancies were addressed through discussion, resulting in a consensus. Patients were then divided into three distinct groups: cylindrical, varicose, and cystic. If a patient exhibits multiple different types of bronchiectasis on HRCT, they will be classified on the basis of the most severe type, such as varicose or cystic type. The severity of bronchiectasis was assessed using the modified Reiff score [6], which considered the involvement and degree of dilation within each lobe. With each lung containing three lobes (including the lingular segment as a separate lobe of the left lung), the scoring for bronchial dilation was as follows: cylindrical = 1, varicose = 2, and cystic = 3. The sum of the scores from all six lobes constituted the modified Reiff score (ranging from 1 to 18). The severity of bronchiectasis was also categorized into three groups based on Reiff scores: Group 1 (mild: 1–6 points), Group 2 (moderate: 7–12 points), and Group 3 (severe: 13–18 points).

### Clinical symptoms and outcome assessment

We attempted to analyze the variations in fundamental demographic data, lung function, clinical symptoms, incidence of pneumonia, and hospitalization among the three groups of patients (cylindrical, varicose, and cystic). Furthermore, we examined the relationship between modified Reiff scores and clinical symptoms, lung function, hospitalization, and pseudomonas pneumonia. Hospitalization and pneumonia data were collected from medical records within one year of the index date. Pneumonia was defined on the basis of alterations in respiratory symptoms, changes in sputum characteristics, and chest radiological findings requiring additional antibiotic treatment. Pseudomonas aeruginosa pneumonia was confirmed through sputum culture results positive for P. aeruginosa in patients treated with antibiotics for pneumonia. Surgical intervention in this study refers to the treatment of hemoptysis resulting from bronchiectasis. Macrolide use in this study encompasses treatment with erythromycin, clarithromycin, or azithromycin for a minimum duration of three months. Hospitalization specifically concentrated on cases with a primary admission diagnosis of pneumonia or bronchiectasis with exacerbation. Mortality data were monitored through medical records until June 2022. Symptom scores were computed, assigning 1 point each for cough, sputum, hemoptysis, and dyspnea, leading to a total score ranging from 1 to 4 points.

### Statistical analysis

All data are expressed as the mean ± standard deviation for continuous variables and numbers (percentage) for categorical variables. Statistical analysis was performed using MedCalc for Windows version 18.10 (MedCalc Software, Ostend, Belgium). Continuous variables were compared using one-way ANOVA tests, whereas categorical variables were assessed using Chi-squared tests. Variables with p-values below 0.1 in the univariate analysis were included in the multivariate analysis. Statistical significance was set at *p* < 0.05. The strength of association is expressed as HR and the associated 95% confidence interval (CI).

## Results

### Baseline characteristics

**Fig. 1:**
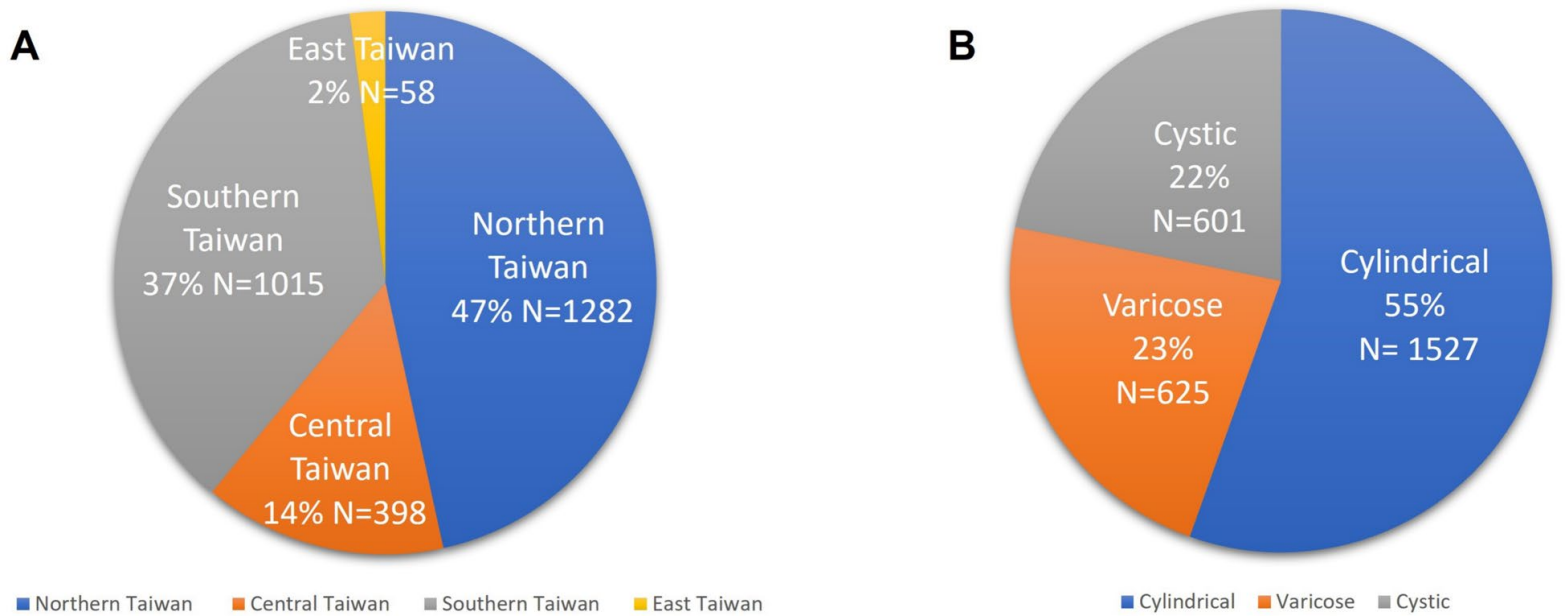
**A** Enrolled patients from North, Central, South, and East Taiwan. **B** Enrolled patients were categorized into cylindrical, varicose, and cystic types of NCFB. *NCFB* Noncystic Fibrosis Bronchiectasis

This study recruited 2753 subjects across 16 sites in Taiwan. The distribution included 1282 patients (47%) from northern Taiwan, 398 patients (14%) from central Taiwan, 1015 patients (37%) from southern Taiwan, and 58 patients (2%) from eastern Taiwan. Among these patients, 1527 patients (55%) were classified as cylindrical type, 625 patients (23%) as varicose type, and 601 patients (22%) as cystic type (Fig. 1).

The baseline characteristics of 2753 patients with NCFB are depicted in Table 1. Cylindrical-type patients showed a higher percentage of males (45.3%) and a smoking history (29.6%) than other types (*p* < 0.001). Cystic patients had the lowest body mass index (BMI) (*p* < 0.001). Cystic-type patients revealed higher clinical symptoms and symptoms scores (2.5, from 1 to 4;* p* < 0.001), including cough (88.2%), sputum (81.5%), hemoptysis (29.8%), and dyspnea (53.7%) (*p* < 0.001). Patients with the cystic type also demonstrated the highest prevalence of comorbidities, including chronic obstructive pulmonary disease (COPD) (40.3%, *p* < 0.001), asthma (21.6%,* p* = 0.043), allergic bronchopulmonary aspergillosis (ABPA) (1.2%, *p* = 0.003), and end-stage renal disease (ESRD) (29.8%,* p* < 0.001), compared with the overall study population. There was no significant relationship between autoimmune disease and bronchiectasis type. However, patients with cystic bronchiectasis showed a higher incidence of prior pneumonia (44.3%) and tuberculosis infections (22.0%) (*p* < 0.001). In addition, they exhibited an increased risk of bacterial pneumonia (45.3%), with notable occurrences of pseudomonas aeruginosa (14.3%) and nontuberculous mycobacterial pneumonia (8.7%) in the whole cohort (*p* < 0.001). In terms of lung function, patients with the cystic type had the lowest FEV1 (1.31 L) and forced vital capacity (FVC) (1.81 L) compared with the other types (*p* < 0.001). Patients with cystic bronchiectasis have higher rates of surgical intervention (*p* = 0.037) and macrolide use (*p* < 0.001). Cystic bronchiectasis patients faced a high risk of hospitalization (0.49) because of bronchiectasis exacerbations (*p* < 0.001). Cystic bronchiectasis patients had a higher severity score according to the modified Reiff score (9.01) (*p* < 0.001).

**Table 1:** Baseline Characteristics of patient with non-cystic fibrosis bronchiectasis between different type on HRCT

	Cylindrical (*n* = 1527)	Varicose (*n* = 625)	Cystic (*n* = 601)	*p*-value
Male (%)	692 (45.3)	226 (36.2)	232 (38.6)	< 0.001
Age (SD)	70.5 (11.9)	70.8 (11.9)	70.9 (11.8)	0.751
BMI (SD)	22.3 (4.1)	21.3 (4.2)	21.2 (4.2)	< 0.001
Smoking (%)	453 (29.6)	124 (19.8)	125 (20.7)	< 0.001
Symptoms (%)				
Cough	1160 (76.0)	514 (82.2)	530 (88.2)	< 0.001
Hemoptysis	267 (17.5)	186 (29.8)	179 (29.8)	< 0.001
Dyspnea	553 (36.2)	261 (41.8)	323 (53.7)	< 0.001
Sputum	1056 (69.2)	480 (76.8)	490 (81.5)	< 0.001
Symptoms scores^&^	1.9 (1.1)	2.3 (1.1)	2.5 (0.9)	< 0.001
Comorbidity (%)				
Hypertension	526 (34.4)	187 (29.9)	204 (33.9)	0.121
Type 2 DM	252 (16.5)	96 (15.4)	97 (16.1)	0.807
CAD	382 (25.0)	126 (20.2)	140 (23.3)	0.054
Liver cirrhosis	28 (1.8)	4 (0.6)	7 (1.2)	0.087
COPD	468 (30.6)	199 (31.8)	242 (40.3)	< 0.001
Asthma	310 (20.3)	102 (16.3)	130 (21.6)	0.043
Allergic Rhinitis	438 (28.7)	147 (23.5)	162 (27.0)	0.051
ABPA	3 (0.2)	1 (0.2)	7 (1.2)	0.003
CKD	109 (7.1)	45 (7.2)	46 (7.7)	0.916
ESRD	267 (17.5)	186 (29.8)	179 (29.8)	< 0.001
Cancer	231 (15.1)	73 (11.7)	79 (13.1)	0.092
Etiology (%)				
Post RT for H& N cancer	24 (1.6)	13 (2.1)	14 (2.3)	0.451
Rheumatoid arthritis	34 (2.2)	12 (1.9)	10 (1.7)	0.691
SLE	6 (0.4)	4 (0.6)	5 (0.8)	0.434
Sjogren’s syndrome	34 (2.2)	13 (2.1)	9 (1.5)	0.561
Prior pneumonia	461 (30.2)	243 (38.9)	266 (44.3)	< 0.001
Prior tuberculosis	179 (11.7)	113 (18.1)	132 (22.0)	< 0.001
Pulmonary function				
FEV1, L (SD)	1.67 (0.66)	1.52 (0.63)	1.31 (0.59)	< 0.001
FVC, L (SD)	2.31 (0.85)	2.09 (0.83)	1.81 (0.71)	< 0.001
FEV1/FVC (%)	74.6 (12.9)	74.4 (12.7)	72.7 (13.9)	0.071
Pneumonia (%)				
Bacteria pneumonia	387 (25.3)	207 (33.1)	272 (45.3)	< 0.001
Pulmonary TB	13 (0.9)	6 (1.0)	2 (0.3)	0.475
Fungal pneumonia	85 (5.6)	31 (5.0)	50 (8.3)	0.243
NTM	81 (5.3)	53 (8.5)	52 (8.7)	0.014
Pseudomonas aeruginosa (%)	62 (4.1)	44 (7.0)	86 (14.3)	< 0.001
Surgical intervention (%)	28 (1.8)	13 (2.1)	22 (3.7)	0.037
Macrolide use	140 (9.2)	87 (13.9)	93 (15.5)	< 0.001
ER visited /Hospitalization (%)	0.21 (0.76)	0.26 (0.74)	0.49 (1.16)	< 0.001
Reiff Score (SD)	3.06 (1.58)	5.69 (2.35)	9.01 (3.57)	< 0.001

*ABPA* Allergic bronchopulmonary aspergillosis, *BMI* Body mass index, *COPD* Chronic obstructive pulmonary disease, *CAD* Coronary artery disease, *CKD* Chronic kidney disease; *ESRD* End stage of renal disease, *ER* Emergency room; *FEV1* Forced expiratory volume in 1 s, *FVC* Forced vital capacity, *GERD* Gastroesophageal reflux disease, *HTN* Hypertension, *H&N* Head and neck, *SD* Standard Deviation, *SLE* Systemic lupus erythematosus, *NTM* Nontuberculous mycobacterium, *RT* Radiotherapy, *T2DM* Type 2 diabetes mellitus, *TB* Tuberculosis

^&^Symptom scores were calculated, assigning 1 point each for cough, sputum, hemoptysis, and dyspnea, resulting in a total score ranging from 1 to 4 points

### Correlation between clinical parameters and outcomes with the modified Reiff score

**Fig. 2:**
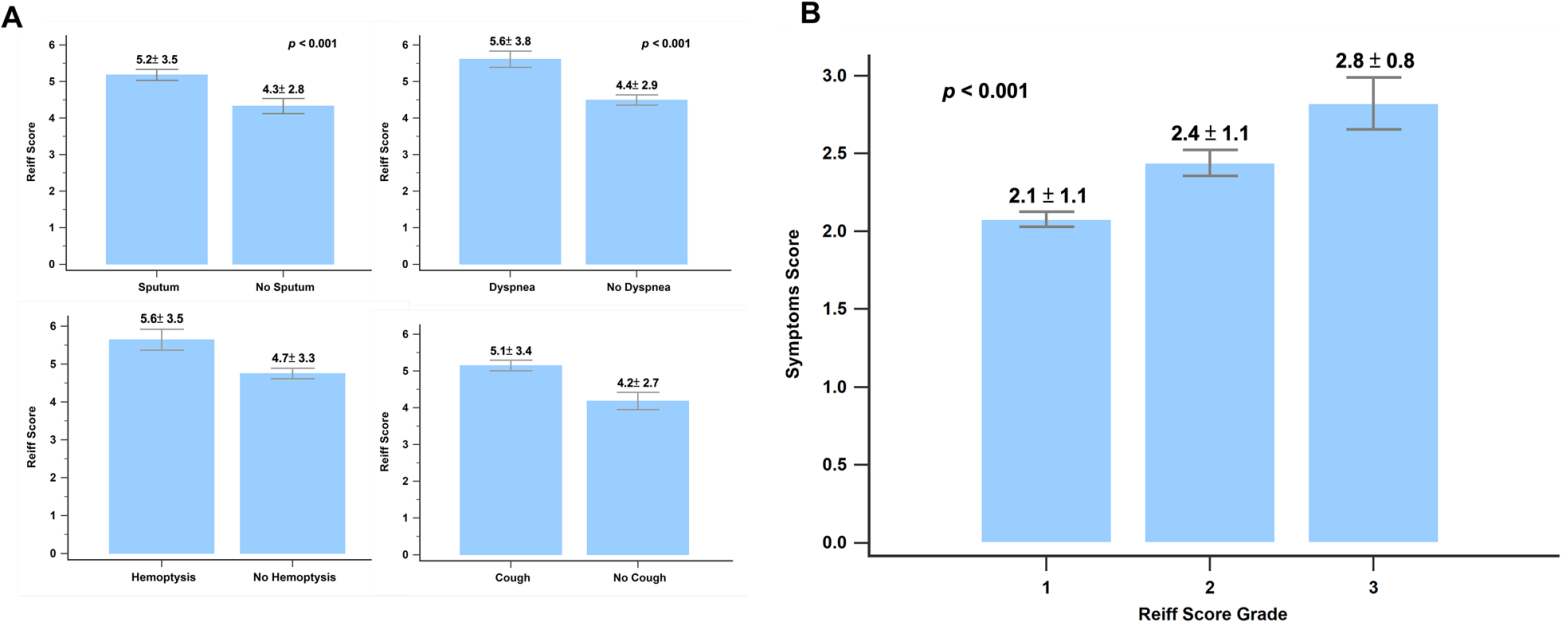
**A** The Reiff scores were compared between patients with and without clinical symptoms (cough, hemoptysis, dyspnea, and sputum). **B** The symptom scores of patients with different severity levels of Reiff scores. Group 1: mild; Group 2: moderate; Group 3: severe

**Fig. 3:**
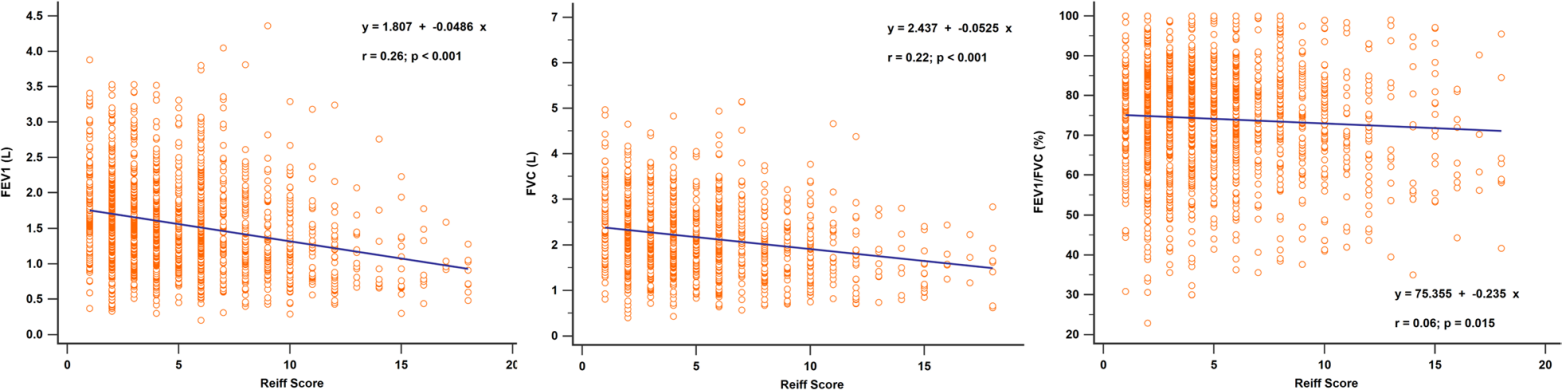
Relationship between the modified Reiff score and FEV1, FVC, and FEV1/FVC. FVC, forced vital capacity; FEV1, forced expiratory volume in 1 s

**Fig. 4:**
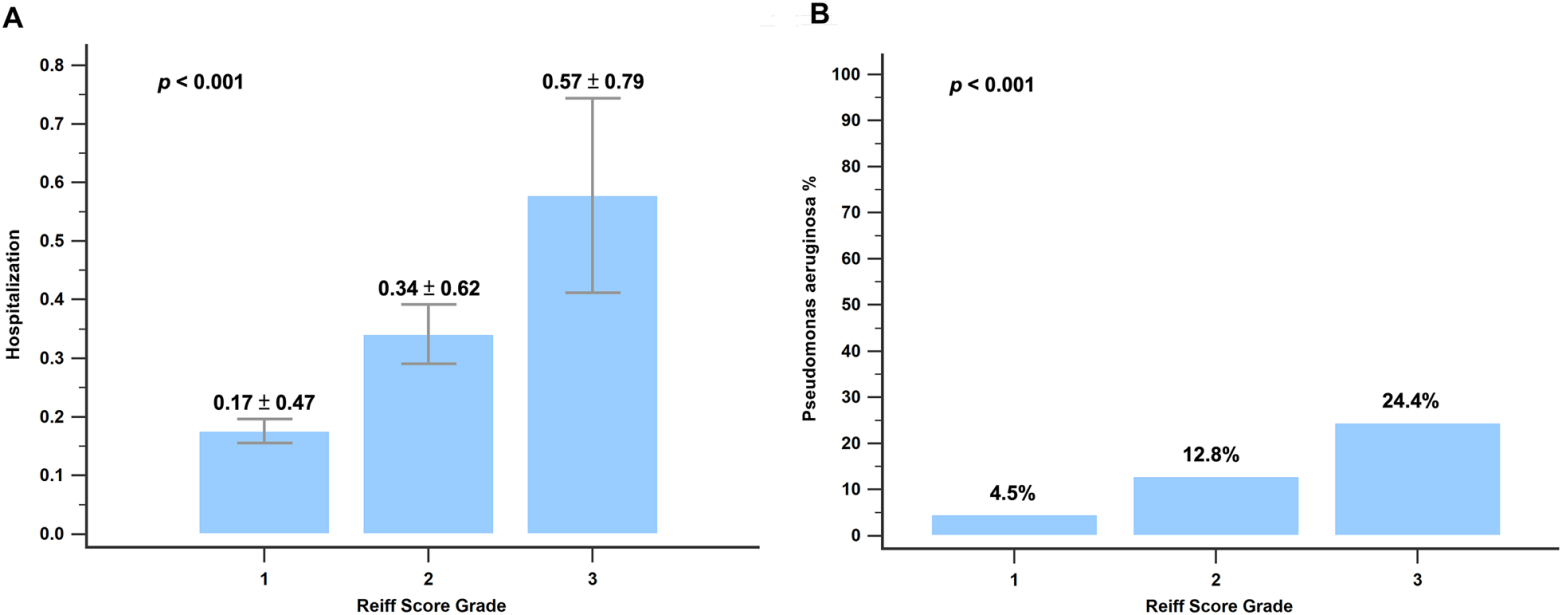
**A** The hospitalization frequency of patients across various severity levels of Reiff scores. **B** The occurrence of Pseudomonas aeruginosa pneumonia among patients at different severity levels of Reiff scores. Group 1: mild; Group 2: moderate; Group 3: severe

Figure 2 shows the correlation between clinical symptoms and the modified Reiff score. Patients experiencing clinical symptoms such as cough, sputum production, hemoptysis, and wheezing showed higher Reiff scores than those without these symptoms. Moreover, the Reiff score was classified into three groups based on severity, and as the severity increased, the symptom scores also increased (*p* < 0.001). As depicted in Fig. 3, there was a considerable decline in forced expiratory volume in the first second (FEV1) (*p* < 0.001), FVC (*p* < 0.001), and FEV1/FVC% (*p* = 0.015) with an increase in the modified Reiff score. Furthermore, as the severity of Reiff scores increased, the hospitalization frequency for bronchiectasis due to acute exacerbations or pneumonia also elevated last year. The incidence of Pseudomonas aeruginosa pneumonia also increased with the severity of Reiff scores (Fig. 4).

### Independent predictors of hospitalization and mortality

We conducted both univariate and multivariate analyses to detect clinical predictors of hospitalization and mortality, as illustrated in Tables 2 and 3. Lower FEV1 (Hazard Ratio (HR) = 0.51, 95% CI 0.37–0.68; *p* < 0.001), pseudomonas aeruginosa pneumonia (HR = 2.24, 95% CI 1.35–3.73; p = 0.002), HR scores (HR = 1.34, 95% CI 1.02–1.76; p = 0.035), and surgical intervention (HR = 2.52, 95% CI 1.02–6.22; p = 0.044) emerged as independent predictors for hospitalization. Furthermore, elderly age (HR = 1.05, 95% CI 1.02–1.09; *p* = 0.005), pseudomonas aeruginosa pneumonia (HR = 3.48, 95% CI 1.46–8.31; *p* = 0.005), and hospitalization due to acute exacerbations (HR = 3.79, 95% CI 2.26–6.37; *p* < 0.001) were identified as independent predictors of mortality.

**Table 2:** Independent of predictor of hospitalization in patients with noncystic fibrosis bronchiectasis

	Univariate			Multivariate model		
	HR	95% CI	*p*-value	HR	95% CI	*p*-value
Age	1.026	1.02–1.03	< 0.001	1.008	0.99–1.02	0.311
BMI	0.951	0.92–0.97	< 0.001	0.998	0.96–1.04	0.911
Symptoms score	1.437	1.28–1.62	< 0.001	1.143	0.96–1.35	0.124
FEV1	0.423	0.32–0.56	< 0.001	0.505	0.37–0.68	< 0.001
Pseudomonas aeruginosa	1.935	1.55–2.42	< 0.001	2.241	1.35–3.73	0.002
Reiff Score	1.106	1.07–1.14	< 0.001	1.342	1.02–1.76	0.035
Surgical intervention	1.815	0.95–3.45	0.069	2.523	1.02–6.22	0.044
Macrolide use	1.359	0.97–1.91	0.075	1.114	0.69–1.79	0.655

*BMI* Body mass index, *FEV1* Forced expiratory volume in 1 s

**Table 3:** Independent Predictors of mortality in patients with noncystic fibrosis bronchiectasis

	Univariate			Multivariate model		
	HR	95% CI	*p*-value	HR	95% CI	*p*-value
Age	1.087	1.06–1.11	< 0.001	1.049	1.02–1.09	0.005
BMI	0.978	0.93–1.03	0.403			
Symptoms score	1.278	1.04–1.57	0.017	0.778	0.57–1.06	0.113
FEV1	0.487	0.28–0.83	0.005	1.072	0.59–1.93	0.861
Pseudomonas aeruginosa	1.635	1.39–1.92	< 0.001	3.479	1.46–8.31	0.005
ER visited last year	1.729	1.51–1.98	< 0.001	1.195	0.68–2.09	0.533
Hospitalization	1.812	1.59–2.06	< 0.001	3.795	2.26–6.37	< 0.001
Surgical intervention	0.981	0.24–4.08	0.978	–	–	–
Macrolide use	0.562	0.24–1.31	0.145	0.408	0.11–1.45	0.166

*BMI* Body mass index, *ER* Emergency room, *FEV1* Forced expiratory volume in 1 s

## Discussion

To the best of our knowledge, this is the first research to comprehensively evaluate the clinical features, symptoms, and disease severity based on the HRCT pattern using the modified Reiff score in Taiwanese patients with NCFB. The present study demonstrated that individuals with cystic-type NCFB showed a greater symptom burden and more comorbidities, such as COPD, asthma, and ABPA. Furthermore, they faced a heightened risk of pseudomonas bacterial pneumonia, hospitalization, and lung function impairment. Cystic-type NCFB patients showed HR scores, which were closely related to poorer lung function, increased symptom severity, higher risk of pseudomonas aeruginosa pneumonia, and higher rates of hospitalization. Our results show that the simplified modified Reiff score aligns with clinical prognostic factors, offering ease of use for clinicians.

The Bronchiectasis Severity Index (BSI) [11] and the FACED score [12] were created based on different clinical parameters to predict the severity and prognosis of bronchiectasis. However, the BSI and FACED scores did not show significant associations with the percent predicted FEV1, sputum purulence, and hospital admissions for bronchiectasis exacerbations. Moreover, these two score systems are relatively complex and need the gathering of more information, making them less user-friendly. As a result, we try to use a simple radiology score to associate with all clinical parameters.

A Korean study of 506 bronchiectasis patients found that obstructive disorders led to worse dyspnea, disease severity, and radiologic findings, with significant reductions in FVC%, FEV1/FVC%, and FEV1% correlating with higher modified Reiff scores (*p* < 0.001) [13]. In a study of 114 bronchiectasis patients, 83.3% exhibited obstructive lung patterns and 68.7% had air trapping, with severe dyspnea correlating with obstructive spirometry findings [14]. The current study not only showed a significant decrease in FEV1 (*p* < 0.001), FVC (*p* < 0.001), and FEV1/FVC% (*p* = 0.015) but also noted an increased symptom burden with higher modified Reiff scores. Our research also indicates that among patients with the same Reiff score but different HRCT patterns, the cystic type of NCFB shows the lowest pulmonary function (FEV1 and FVC) and higher symptoms burden compared with other HRCT patterns (cylindrical or varicose). Previous studies have established a connection between sputum purulence and bronchiectasis severity [15]. Airflow limitation is commonly found in advanced stages of bronchiectasis [16]. Studies show a connection between airflow obstruction in bronchiectasis and structural abnormalities detected on CT scans [12, 17].

Chalmers et al. studied bronchiectasis patients from 10 centers across Europe and Israel over 5 years, finding that exacerbations, associated with more severe disease, reduced quality of life, and higher mortality, were predicted by pseudomonas aeruginosa infections, lower FEV1, radiologic severity, and concurrent COPD [18]. The current study found that lower FEV1, the presence of pseudomonas aeruginosa pneumonia, and HR scores emerged as independent predictors for hospitalization due to acute exacerbation of bronchiectasis. The incidence of pseudomonas aeruginosa pneumonia also increased with the severity of modified Reiff scores. Our research also discovered that advanced age, pseudomonas aeruginosa pneumonia, and hospitalization due to acute exacerbations of bronchiectasis were independent predictors of mortality. This study included NCFB patients from 16 sites in Taiwan, with a follow-up period of up to 3 years. In these two studies concentrating on bronchiectasis across two distinct ethnic groups, a consistent pattern emerged: as the modified Reiff score increased, it was associated positively with clinical symptoms burden, decreasing lung function, increased risk of pseudomonas pneumonia, higher rates of hospitalization due to acute exacerbations, and ultimately mortality. Hence, using a simplified Reiff score can efficiently stratify the severity of NCFB across several ethnic populations.

This study also demonstrated a high incidence of COPD among patients with cystic NCFB. Recent studies show that bronchiectasis and COPD coexist in 20–60% of cases [19–21]. The coexistence of these diseases can lead to an increased symptom burden and a poorer prognosis compared with COPD or bronchiectasis alone [22]. Patients with cystic-type NCFB not only revealed a high risk of prior pneumonia and a history of tuberculosis infections but also had a higher incidence of ABPA. Yang et al. found that COPD, previous pulmonary tuberculosis, and nontuberculous mycobacterial disease raised aspergillosis risk in bronchiectasis patients [23]. Ma et al. also reported that asthmatic patients with bronchiectasis had more frequent asthma exacerbations [24]. The current study observed that individuals with cystic NCFB had a higher incidence of comorbid asthma. This may also explain why cystic NCFB is more prone to acute exacerbations. Furthermore, our study highlighted the increased risk of pseudomonas aeruginosa infection in patients with cystic NCFB. Wang et al. discovered that pseudomonas aeruginosa colonization associates with greater lung involvement and a higher risk of exacerbations requiring hospitalization [25]. Lee et al. reported that being underweight is linked with heightened mortality among individuals with bronchiectasis [26]. In this study, it was also observed that cystic-type NCFB was associated with the lowest BMI and poor clinical prognosis. To the best of our knowledge, our study is the first to emphasize that cystic-type NCFB is connected with high comorbidities and a high risk of pseudomonas aeruginosa pneumonia, beyond the scope of modified Reiff scores alone.

This study had several limitations that should be mentioned. First, its retrospective design may introduce selection bias. Although bronchiectasis can present differently across geographical regions, our results align with those of previous studies in Europe and Israel [18]. Second, due to constraints in healthcare data, identifying the etiology of bronchiectasis was limited. Third, patients were recruited in June 2020, and mortality data were obtained by June 2022. This timeframe may have resulted in some subjects being censored before the 3-year mark, which could impact the accuracy of the estimated 3-year mortality rate. Finally, due to the retrospective nature of the study, the microbiological survey and timing were not strictly regulated, potentially resulting in an underestimation of the incidence of pseudomonas aeruginosa pneumonia. To confirm these findings, a prospective registry is essential.

## Conclusion

The current study represents the first large cohort study of Taiwanese NCFB patients, aiming to confirm the modified Reiff score’s relationship with clinical symptoms, pulmonary function, hospitalization due to acute exacerbations of bronchiectasis, and the incidence of pseudomonas aeruginosa pneumonia. Our results suggest that the modified Reiff score serves as a simplified radiological tool for clinical decision-making. In addition, cystic-type NCFB is associated with more comorbidities and poorer clinical prognosis.

## Data Availability

The corresponding author is willing to provide the datasets used and/or analyzed in the current study upon reasonable request.
